# The effect of ageing on human lymphocyte subsets: comparison of males and females

**DOI:** 10.1186/1742-4933-7-4

**Published:** 2010-03-16

**Authors:** Jun Yan, Judith M Greer, Renee Hull, John D O'Sullivan, Robert D Henderson, Stephen J Read, Pamela A McCombe

**Affiliations:** 1 The University of Queensland, UQ Centre for Clinical Research, Royal Brisbane & Women's Hospital, Brisbane, Australia; 2 Wesley Research Institute, Wesley Hospital, Brisbane, Australia; 3 Department of Neurology, Royal Brisbane and Women's Hospital, Brisbane, Australia

## Abstract

**Background:**

There is reported to be a decline in immune function and an alteration in the frequency of circulating lymphocytes with advancing age. There are also differences in ageing and lifespan between males and females. We performed this study to see if there were differences between males and females in the frequency of the different lymphocyte subsets with age.

**Results:**

Using flow cytometry we have examined different populations of peripheral blood leukocytes purified from healthy subjects with age ranging from the third to the tenth decade. We used linear regression analysis to determine if there is a linear relationship between age and cell frequencies. For the whole group, we find that with age there is a significant decline in the percentage of naïve T cells and CD8^+ ^T cells, and an increase in the percentage of effector memory cells, CD4^+^foxp3^+ ^T cells and NK cells. For all cells where there was an effect of ageing, the slope of the curve was greater for men than for women and this was statistically significant for CD8^+^αβ^+ ^T cells and CD3^+^CD45RA^-^CCR7^- ^effector memory cells. There was also a difference for naïve cells but this was not significant.

**Conclusion:**

The cause of the change in percentage of lymphocyte subsets with age, and the different effects on males and females is not fully understood but warrants further study.

## 1. Introduction

It is known that there is a loss of lymphoid tissue [1] and a decline in the function of the human immune system with increasing age [2-4]. This decline, sometimes termed "immunosenescence" [5,6], has been implicated in the increased susceptibility of aged people to a number of diseases, including cardiovascular disease [7,8], autoimmune disease and malignancy, and to impairment of response to vaccination and infection [9,10]. Males have a shorter lifespan than females and thus may be more susceptible to the effects of aging [11]. The immune system of males also has differences from the immune system of females [12,13]. However, little is known about whether males and females show differences in the effects of aging on the immune system. We have been particularly interested in the percentages of cells in peripheral blood in older age groups, because of our studies of the peripheral immune response to stroke, [14] which affects an older age group.

Current studies indicate that impaired immune function with age is associated with alterations in cell numbers, and also, in humans and in rats, with decreased T cell activation and proliferation [15-18]. With ageing in humans there is a decline in the number of naïve cells, an increase in the ratio of memory to naïve cells [4], the number of memory T cells [19,20], and the ratio of CD4^+ ^to CD8^+ ^cells [21] and an increase in the percentage of NK cells [22] although the function of NK cells declines. Less is known about the changes in immunoregulatory T cells (Treg) with age, but the number of CD4^+ ^Treg cells [23-25] and the frequency of CD8^+ ^Treg cells [26] have been reported to increase with age. However, there are suggestions in mice that CD4^+^CD25^- ^effector cells become incompetent with age [27].

The mechanisms involved in the decline in immune function with age are not fully understood. These changes are often ascribed to changes in the length of telomeres, although this is controversial [28]. Even though T cells can use telomerase to maintain the length of telomeres during cell proliferation, with ageing there is a reduction of the length of their telomeres due to loss of telomerase activity [29,30]. With increasing age, telomerase activity is better preserved in NK cells than in CD8^+ ^T cells [31]. In monkeys, the loss of naïve cells is correlated with loss of telomere length [32]. Other proposed mechanisms of immunosenescence are microsatellite instability due to abnormal DNA repair [33] or to age-related epigenetic changes [34]. It is thought that immunosenescence is a consequence of chronic antigenic stress [35,36]. Cytomegalovirus infection appears to contribute to immunosenescence [37] by chronic stimulation and activation of CD8^+^ cells [38].

To investigate the effects of age and gender on human lymphocyte populations, we studied lymphocyte subsets and their expression of activation markers in peripheral blood in healthy people above the age of 21, and analyzed this according to gender.

## 2. Subjects and Methods

### 2.1. Subjects and blood collection

The procedures involved in the study were approved by Royal Brisbane and Women's Hospital Health Service District Office of the Human Research Ethics Committee and The Medical Research Ethics Committee, The University of Queensland, Brisbane, Australia. Blood (50 ml) was collected from healthy volunteers by venipuncture. We regarded subjects as being healthy if they had no acute illness, and were on no medication other than anti-hypertensive medication, and had no serious prior illnesses. We did not investigate whether the subjects had previous infection with Epstein Barr virus or cytomegalovirus. The age and sex distribution of the subjects are summarized in Table 1.

**Table 1:** Age and sex of participants in the study

	Number of subjects		
Age group	Total	Male	Female
20's	**12**	**5**	**7**
30's	**14**	**7**	**7**
40's	**12**	**6**	**6**
50's	**14**	**7**	**7**
60's	**12**	**6**	**6**
70's	**9**	**3**	**6**
>80	**7**	**3**	**4**
Total	**80**	**37**	**43**

### 2.2. Purification of PBL and staining for flow cytometry

Blood was separated by density gradient centrifugation through LymphoSep (MP Biotechnologies). Peripheral blood leukocytes (PBL) were then isolated and washed twice with PBS containing 1% supreme serum, counted, and the concentration of cells (1 × 10^7^per ml) of suspension was determined. All the data generated by flow cytometry was from freshly purified PBL. Antibodies used for staining were against CD3, CD4, CD8, CD20, CD25, CD45RA, CD69, αβTCR, γδTCR and CCR7 (all from BD, conjugated either to FITC, PE, PerCP or APC) and FoxP3 (from eBioScience, PE-conjugated). Fluorochrome-conjugated isotype-matched antibodies were used as negative controls. For surface staining, PBL (1 × 10^6 ^cells in 100 μl PBS containing 1% serum and 0.1% NaN_3_) were incubated with 1 μg antibody in the dark at 4°C for 30 min and then washed twice. For detecting regulatory T cells, PBL were firstly incubated with anti-CD4-FITC and anti-CD25-APC antibodies, then were fixed, permeabilized, incubated with anti-FoxP3-PE or appropriate isotype control and washed 3 times. Cells were analyzed on a four-colour flow cytometer (FACSCalibur, BD), with gating on the total lymphoid and monocyte populations, as previously described [14]. Samples were obtained and studied individually. For consistency, for each flow cytometry analysis we used the standard calibration beads (BD) to set the forward scatter and side scatter and PMT voltage. The compensation was then adjusted by single staining PBL cells (in particularly, cells from each sample were stained with FITC,/PE/PerCP/APC/Alexa 46 in each experiment). For the experimental samples, a corresponding isotype control was used to set gates, or positive/negative cell populations.

### 2.3. Statistical analysis

To analyse the number of T cells, B cells, activated T cells and activated B cells, first we gated on the lymphoid cell population. For naïve, effector or central memory cells, we first gated on the CD3 cell population. CD4/CD8 cell population was gated for analysis of alpha beta TCR/gamma delta TCR cells. For Treg analysis, we gated on the CD4 cell population. To investigate whether there was a linear relationship between age in years and the percentage of cells with different cell surface markers, we performed linear regression analysis. To determine whether there was a statistically significant difference between men and women, we compared the slope of the curves, using Graphpad prism. Data are expressed as the mean ± S.D. Statistical significance between groups was evaluated using nonparametric Kruskal-Wallis test within One-way ANOVAL in GraphPad. The statistical data was considered as significant if *P *< 0.05.

## 3. Results

**Figure 1:**
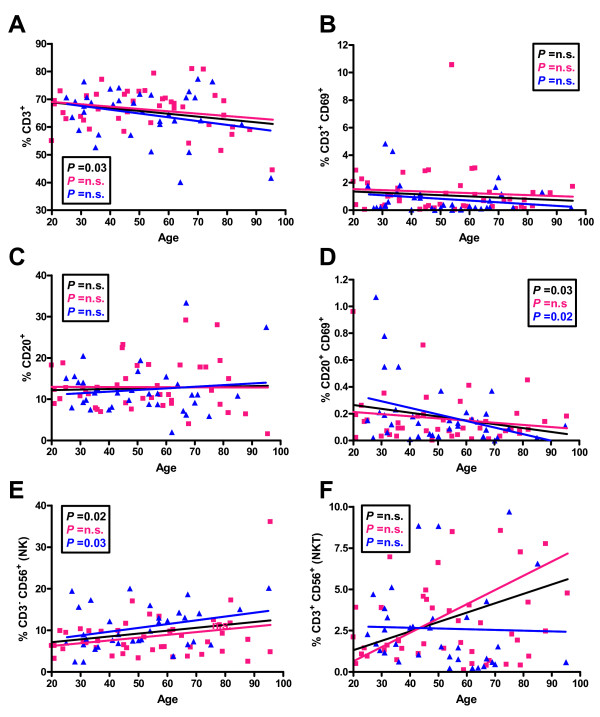
**Distribution of percentages of PBL from individuals of different ages bearing different cell markers**. The cell markers that PBL were stained for are shown on the Y axis. The linear regression results for all individuals (black line), males (blue line) and females (pink line) and the relevant P values are shown on the graphs. n.s. = not significant.

The results of linear regression analysis for CD3 (T cells/NKT cells), CD20 (B cells) and CD56 (NK/NKT cells) are shown in Figure 1. In the CD3^+ ^cells population (Figure 1A) there was a significant decline in cell frequency that was significant for the combined group of males and females, but not for males and females alone. For the activated CD3^+^CD69^+^ cell population (Figure 1B) and the CD20+ B cell population (Figure 1C) there were no significant differences with age. There was a significant decline in the percentage of activated CD69^+ ^B cells with age in males, but not females (Figure 1D), and a significant increase in the percentage of NK cells with age in males (Figure 1E). There were no significant changes with age in the NKT cell population (Figure 1F).

**Figure 2:**
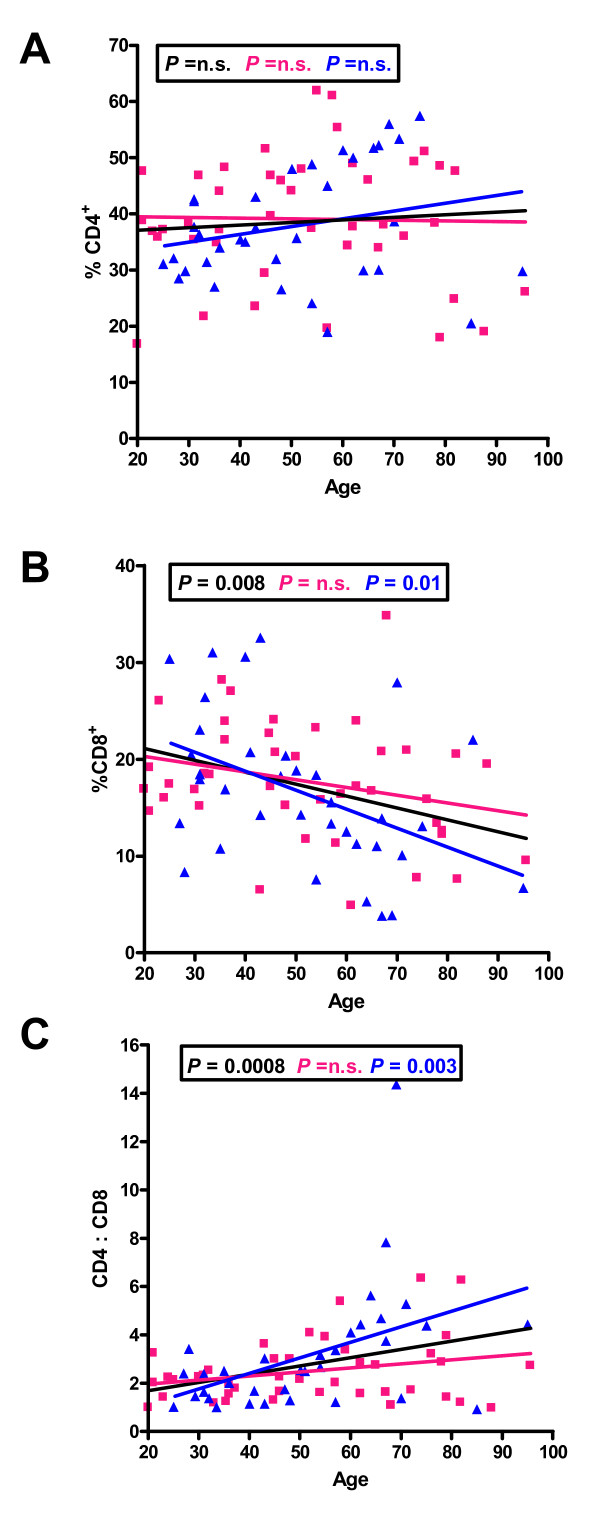
**Distribution of percentages of lymphocytes from individuals of different ages bearing CD4 (A) or CD8 (B) and the change in the CD4:CD8 ratio with aging (C)**. The linear regression results for all individuals (black line), males (blue line) and females (pink line) and the relevant P values are shown on the graphs. n.s. = not significant.

When CD3^+ ^cells were further subdivided into CD4^+ ^and CD8^+ ^T cells (Figure 2), there were no significant changes in the frequency of CD4^+^ cells with age (Figure 2A). There was a significant decrease in the percentages of CD3^+^CD8^+ ^T cells in males with aging (Figure 2B), and with aging there was an increase in the ratio of CD4:CD8 T cells in males (Figure 2C).

**Figure 3:**
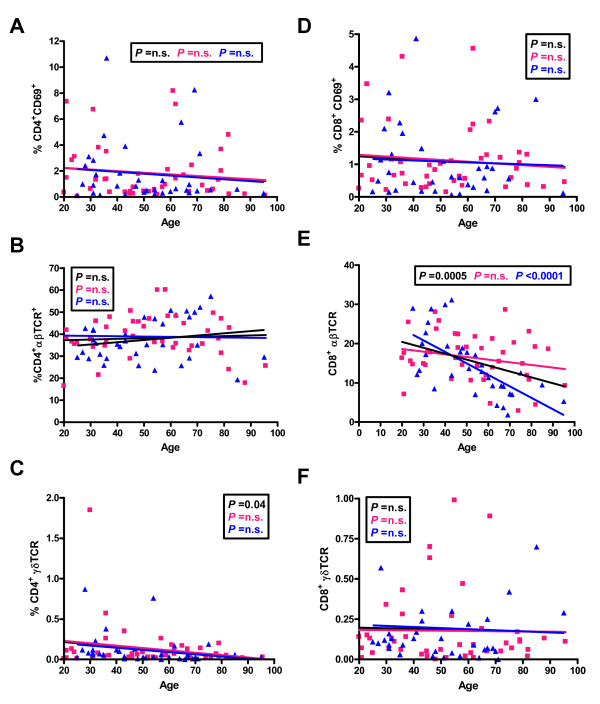
**Distribution of percentages of activated CD4 (A) or CD8 (D) T lymphocytes, and those carrying either the αβTCR (B and E) or γδTCR (C and F) from individuals of different ages**. The linear regression results for all individuals (black line), males (blue line) and females (pink line) and the relevant P values are shown on the graphs. n.s. = not significant.

There was no change in the frequency of activated CD4^+^CD69^+^ cells with age (Figure 3A) or of CD8^+^CD69^+^ cells (Figure 3D). For CD4^+^ TCRαβ^+^ T cells there was no significant change with age (Figure 3B) but for CD8^+^ TCRαβ+ cells there was a significant decline with age that was significant in males but not females (Figure 3E). For TCR γδ cells there was no significant change with age (Figure 3c and 3F).

**Figure 4:**
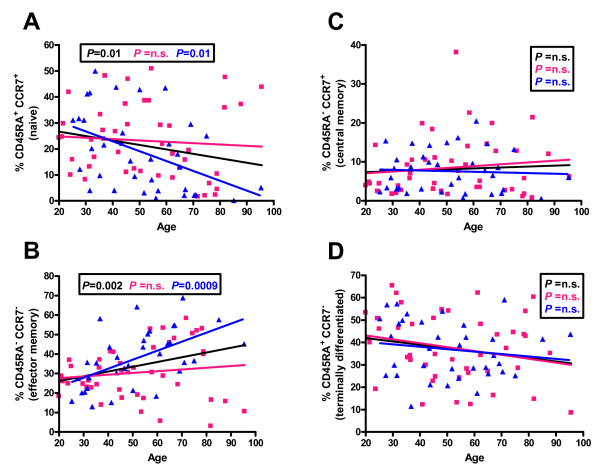
**Distribution of percentages of naïve (CD45RA^+^CCR7^+^) (A), effector memory (CD45RA^-^CCR7^-^) (B), central memory (CD45RA^-^CCR7^+^) (C), or terminally differentiated (CD45RA^+^CCR7^-^) (D) CD3^+^cells from individuals of different ages**. The linear regression results for all individuals (black line), males (blue line) and females (pink line) and the relevant P values are shown on the graphs. n.s. = not significant.

The CD3^+ ^cells were also subdivided on the basis of CD45RA and CCR7 expression into CD3^+^CD45RA^+^CCR7^+ ^naïve cells, CD3^+^CD45RA^-^CCR7^- ^effector memory cells, central memory cells, and terminally differentiated subtypes (Figure 4). With aging, there was a significant decrease in the naïve population that was significant in males but not females (Figure 4A). There was no significant change with age for effector memory cells (figure 4C). There was a significant increase in effector memory cells with age, and this was highly significant in males but not females (Figure 4B). There was no significant change with age in terminally differentiated cells (Figure 4D).

**Figure 5:**
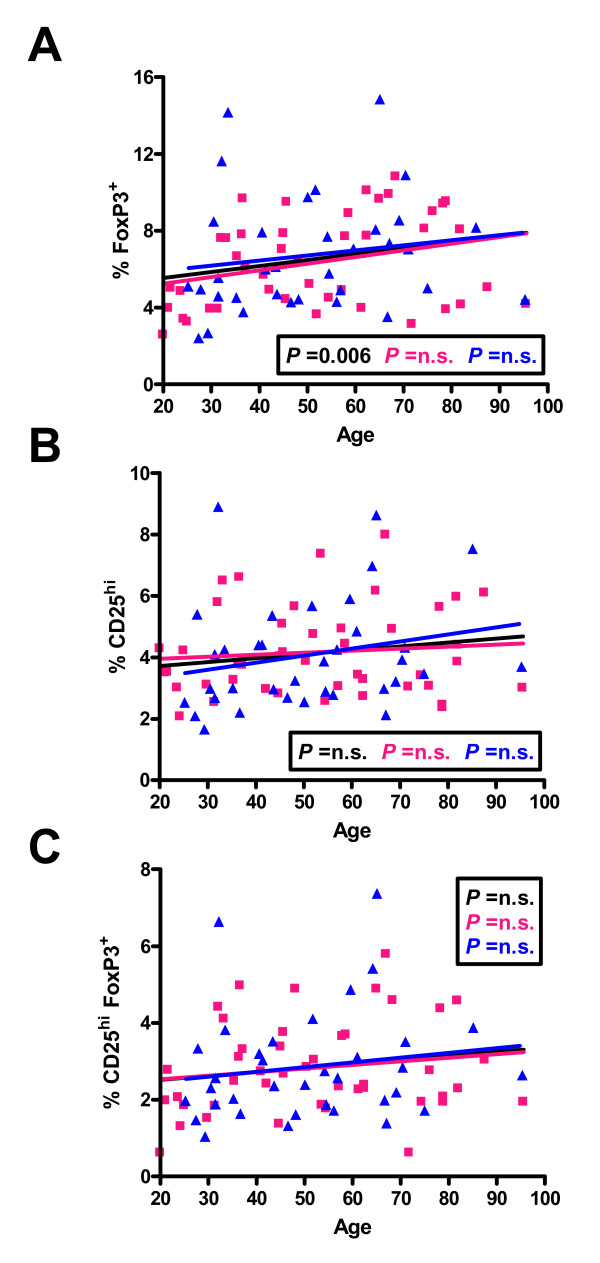
**Distribution of percentages of lymphocytes from individuals of different ages bearing CD4 and Foxp3 (A), CD4 and high levels of CD25 (B), and those CD4+ cells that were positive for both high levels of CD25 and Foxp3 (C)**. The linear regression results for all individuals (black line), males (blue line) and females (pink line) and the relevant P values are shown on the graphs. n.s. = not significant.

As shown in Figure 5, we also analyzed Cd3^+^CD4^+^ cells on the basis of expression of CD25 and Foxp3. Foxp3 is a marker of regulatory T cells (Treg cells), but also appears to be increased transiently in most activated human CD4^+ ^T cells [39,40]. When CD4^+ ^T cells were analyzed, there was a no significant change with age in the percentage of cells expressing Foxp3 (Figure 5A), nor in the percentage of cell that were CD25^hi ^or the percentage of CD25^hi ^cells that were CD25^hi^Foxp3^+^, suggesting that the percentage of Treg cells does not change markedly with age.

To compare the effects of aging in males and females, we directly compared the slopes of the curves for males and females. This is shown in Table 2. There were significant differences in the slopes of the curves for the CD4^+^: CD8^+^ ratio, for CD8^+ ^αβ^+ ^T cells and central memory cells, as shown in Table 2.

**Table 2:** Comparison of results of linear regression analysis of male and female subjects

	Slopes from Best fit values		Are lines different?
	Female	Male	p =
CD4	**-0.01286 ± 0.08489**	**0.1511 ± 0.09574**	**0.209**
CD8	**-0.08004 ± 0.04473**	**-0.2004 ± 0.06368**	**0.119**
CD4:CD8	**0.01671 ± 0.009527**	**0.06488 ± 0.02034**	0.025*
CD4CD69	**-0.01249 ± 0.01596**	**-0.01146 ± 0.02153**	**0.969**
CD4αβTCR	**-0.01396 ± 0.07185**	**0.1150 ± 0.09017**	**0.265**
CD4γδTCR	**-0.002933 ± 0.002135**	**-0.002525 ± 0.001673**	**0.8877**
CD8CD69	**-0.005258 ± 0.007774**	**-0.001859 ± 0.01032**	**0.7908**
CD8αβTCR	**-0.06798 ± 0.04533**	**-0.2926 ± 0.05329**	0.002*
CD8γδTCR	**-0.0001585 ± 0.001757**	**-0.0002358 ± 0.002729**	**0.9803**
CD20	**-0.002255 ± 0.04478**	**0.02262 ± 0.03418**	**0.4128**
CD20CD69	**-0.001613 ± 0.001390**	**-0.004036 ± 0.001982**	**0.3102**
CD3	**-0.08411 ± 0.05615**	**-0.1377 ± 0.07982**	**0.5768**
CD3CD69	**-0.007351 ± 0.01303**	**-0.01537 ± 0.01006**	**0.6483**
Naïve	**-0.05219 ± 0.1052**	**-0.3391 ± 0.1251**	**0.0847**
terminal differentiation	**-0.1740 ± 0.1124**	**-0.1705 ± 0.1217**	**0.9837**
effector memory	**0.08792 ± 0.1042**	**0.4715 ± 0.1100**	0.0156*
central memory	**0.04627 ± 0.05656**	**0.002732 ± 0.04994**	**0.5846**
CD4^+^CD25^hi^	**0.006544 ± 0.01064**	**0.01096 ± 0.01729**	**0.8217**
CD25^hi^ Foxp3	**0.009368 ± 0.009073**	**0.001000 ± 0.01449**	**0.6129**
CD4^+^Foxp3	**0.03464 ± 0.01714**	**0.03181 ± 0.02767**	**0.9281**
CD25Foxp3	**0.003791 ± 0.006090**	**-0.01047 ± 0.01352**	**0.3054**
CD3^-^CD56^+^	**0.06688 ± 0.03635**	**0.07099 ± 0.04662**	**0.9454**
CD3^+^CD56^+^	**0.08633 ± 0.03592**	**-0.001413 ± 0.02298**	**0.073**

Asterisks denote statistically significant results

## 4. Discussion

Ageing is known to have effects on immune function and on the percentages of circulating lymphocytes. Ageing has different effects in males and females with males having a shorter life-span than females [11], so we have investigated whether males and females show different effects of ageing in human peripheral blood lymphocytes. In this study we did not address the functional capacity of these cells. We examined the effects of age on CD3^+ ^lymphocytes expressing CD4, CD8, CD69, CCR7, CD45RA and CCR7, on CD20 B cells, on T regulatory cells, defined by expression of CD25 and foxp3, and on NK cells. The older subjects were healthy in having no active diseases and having no serious previous illnesses. We did not perform serology to estimate prior exposure to CMV or EBV, although we note that chronic infection with these viruses has been proposed to play a role in immunosenescence [41] and that very old subjects have large numbers of T cells reactive with CMV [42].

By linear regression analysis we found no significant changes in the percentage of CD3^+ ^T cells or CD20^+ ^B cells with age, although we did find a significant decrease in activated B cells with age in males. In mice there is known to be a reduction in production of B cells with aging [43] although this is compensated in part by increased lifespan of B cells [44-46]. The age-related impairment of B cell development is associated with impaired V-DJ heavy chain gene recombination [47,48] and also related with changes in the expression and activity of the basic helix-loop-helix proteins E2A-encoded E12 and E47 transcription factors, which help the expression of immunoglobulin heavy chain by binding to the immunoglobulin heavy chain enhancer.

We found an increase in the proportion of NK cells. This is also consistent with previous studies [22] that find increased numbers but reduced functional capacity of NK cells. There are reports of increased NK cells in bone marrow, and indeed these cells are thought to contribute to a decrease in B cell precursors in old age, by inhibiting E2A protein and E47 transcription factors [49]. It has been suggested that ageing is a state when the innate immune system prevails over the adaptive immune system. However, there is also a decline in NK cell activity, seen also in rats, which is more pronounced in males than females [18].

There was no significant change in the percentage of CD4^+ ^cells but there was a decline in the percentage of CD8^+ ^cells and an increase in the ratio of CD4:CD8 cells, as has been previously reported [21]. The decline in CD8^+^ cells was more apparent in the TCRαβ than in the TCRδγ subsets. There was a significant decline in the percentage of CD3^+^CD45RA^+^CCR7^+ ^naïve cells and an increase in the percentage of CD3^+^CD45RA^-^CCR7^- ^effector memory cells with age. The increase in effector memory cells has been suggested to be due in part to chronic antigenic stimulation [38,50].

We also studied T regulatory cells. Previously these cells have been identified as CD4^+^CD25^hi ^according to high constitutive surface expression of interleukin 2 receptor alpha chain CD25 on CD4^+ ^T cells [51,52]. Recently transcription factor Foxp3 has been recognized as the most specific marker of T regulatory cells [53,54], although Foxp3 also appears to be increased in most activated human CD4^+ ^T cells [39,40]. We measured the CD4^+^CD25^hi^Foxp3^+ ^cells, CD4^+^CD25^hi ^cells and CD4^+^CD25^hi^foxp3^+^ cells, and found that although the percentages of Foxp3^+ ^cells increased with age in the total CD4^+ ^population, there were no significant changes in the percentage of CD4^+ ^T cells that were both CD25^hi ^and Foxp3^+ ^with age. In humans, some previous studies have found an increase in Treg cells with age [23,24]. Others have found increased CD4^+^CD25^+ ^cells with age, but no increase in CD4^+^CD25^hi ^cells with age, and attributed the increase in CD4^+^CD25^+ ^cells to an increase in cells with intermediate rather than high levels of expression of CD25 [25]. We did not measure the functional capacity of these cells, and acknowledge that there are studies showing that the functional capacity of human CD4^+ ^Treg cells declines with age [55].

The reason for gender differences in immunosenescence are a matter for speculation. There are known to be gender differences in the immune system of males and females. In males the total lymphocyte count is similar to that in females but the percentage of T cells within the lymphocyte population is lower [56,57]. There are differences in the function of the immune system in males and females [12,13], and this is probably contributes to the different ability of males and females to deal with infections, and the different prevalence of autoimmune disease in males and females [58]. Generally, females produce more vigorous humoral and cellular immune responses than males [59,60], shown in mice as an augmented responses to different antigens [61], ability to reject allografts more rapidly that males [62], and in mice and humans by better in vitro responses to mitogens [60,63] and relative resistance to the induction of immune tolerance [64] There is a superior ability of female mice to combat various infections, including with Leishmania and amebic infection with liver abscess [65], which is thought to be due to due to sex difference in Th1 and Th2 responses [66]. Moreover, in Wistar rats infected with *Trypanasoma cruzi*, there is less parasitaemia in females than males [67].

In the current study we are looking at the differences in immunosenescence between males and females. The changes that we observed with ageing were more apparent in males, although this was statistically significant only for CD8^+^ alpha beta T cells and for effector memory cells. This observation of gender differences in ageing in the immune system is not unique to the immune system. In the heart, there is loss of myocardial mass in men but not in women [68]. Loss of volume in the brain with ageing occurs to a greater extent in men than in women [69]. We note that in all animal species there are gender differences in the effects of ageing, and for humans and for species with species with XY chromosomes, ageing had greater effects in males [11]. Some of this may be due to the effects of hormones. For example, estrogen stimulates c-myc which stimulates telomerase, which could have an anti-ageing effect [70]. Another recent theory relates to the possibility that the evolutionary needs of females and males are different and that mitochondria are better adapted to females than males cells [71]. Our study suggests that there can be differences in immunosenescence between males and females and that this is worth further study.

## Competing interests

The authors declare that they have no competing interests.

## Authors' contributions

JY performed the analysis, JG supervised the FACS analysis, RH was responsible for recruiting and consenting subjects, JO, RH and SR contributed to the recruitment of subjects and to the development of the study, PM has overall responsibility for the project and for writing the paper. All authors read and approved the final manuscript.
